# Epidemiological Survey and Phylogenetic Characterization of* Cysticercus tenuicollis* Isolated from Tibetan Pigs in Tibet, China

**DOI:** 10.1155/2017/7857253

**Published:** 2017-05-18

**Authors:** Houqiang Luo, Hui Zhang, Kun Li, Mujeeb Ur Rehman, Khalid Mehmood, Yanfang Lan, Shucheng Huang, Jiakui Li

**Affiliations:** ^1^College of Veterinary Medicine, Huazhong Agricultural University, Wuhan, China; ^2^College of Animal Science, Wenzhou Vocational College of Science & Technology, Wenzhou, China; ^3^University College of Veterinary & Animal Sciences, The Islamia University of Bahawalpur, Bahawalpur, Pakistan; ^4^Tibet Agriculture and Animal Husbandry College, Linzhi, Tibet, China

## Abstract

*Cysticercus tenuicollis*, commonly known as “water bell,” is a larva of* Taenia hydatigena*, which is the most significant parasite of pigs. However, until now very few information is available regarding the prevalence and genetic characterization of the* Cysticercus tenuicollis* in Tibetan pigs. Therefore, the aim of this study was to investigate the prevalence and phylogenetic analysis of* Cysticercus tenuicollis* in Tibetan pigs. For this purpose, the COX2 gene of* Cysticercus tenuicollis* was amplified and sequenced for the first time in Tibetan pigs. The overall prevalence of* Cysticercus tenuicollis* was 43.93% in Tibetan pigs, with further distribution of 42.86% in 2014 and 45.35% in 2015. In Tibetan male and female pigs, the prevalence of* Cysticercus tenuicollis* was 43.39% and 44.56%, respectively. The prevalence of* Cysticercus tenuicollis* in different growing stages (juveniles, subadults, and adults) varied from 30.20% to 63.79%. The phylogenetic analysis of the* Cysticercus tenuicollis* isolates showed very close resemblance to 16 reference strains, isolates from Gansu, Hunan, and Sichuan provinces of China. To the best of our knowledge, this is the first report on the prevalence and genetic characterization of* Cysticercus tenuicollis* derived from Tibetan pigs. The data of present study provides baseline information for controlling cysticerci infections in pigs in Tibetan Plateau, China.

## 1. Introduction

*Cysticercus tenuicollis* is a bubble-like metacestode of* Taenia hydatigena* that invades the abdominal cavity and liver of pigs, dogs, and other carnivores [1–3]. This parasite is generally associated with the significant tissue damage in liver, lungs, and other organs of pigs, cattle, sheep, and other types of livestock [4–7]. The parasite can infect the pigs of any age and can not only shorten the feed intake but also hinder the growth and development of animals. Therefore, considerable tissue damage of liver or other organs by this parasite may perhaps lead to the death or serious economic losses to the pig industry [8].

The prevalence of* Cysticercus tenuicollis* in domestic pigs has been reported from certain parts of China. Previously, a low prevalence of* Cysticercus tenuicollis* (1.08%) in pigs was reported from Hunan province of China, whereas the high prevalence rates (42.43% to 35.98%) were observed in Fujian and Yunnan [9–11]; additionally, regional distribution of* Cysticercus tenuicollis* infection in pigs has been also reported from Sichuan and Yunnan [12]. However, no report has been available about the prevalence of* Cysticercus tenuicollis* infection in Tibetan pigs.

Tibetan pigs are relatively ancient original indigenous breed, a rare plateau type of pigs in the world, and are the only high altitude pasture pigs in China. Tibetan pigs are mainly distributed in the Qinghai-Tibetan Plateau in China (Qinghai, Sichuan, Yunnan, and eastern Tibet region) [13]. With high proteins and rich amino acids, the Tibetan pig meat is an important source of income for Tibetans nomads [13]. Due to outdoor grazing of Tibetan pigs round the year with the main intermediate hosts (domestic dogs and pi-dog) of the* Cysticercus tenuicollis*, pigs can be exposed to this infection. Furthermore, due to the lack of knowledge at herdsmen level and no special protection, awareness or insect repellent program of the parasitic infection in study area enhances the risk of this infection in ancient original indigenous breed and can lead to significant economic losses.

To date, no report is available about the prevalence and genetic characterization of* Cysticercus tenuicollis* in Tibetan pigs, China. The current study was aimed at investigating the prevalence and genetic characterization of cytochrome oxidase subunit 2 (COX2) derived from* Cysticercus tenuicollis* in Tibetan pigs.

## 2. Materials and Methods

### 2.1. Study Site

**Figure 1:**
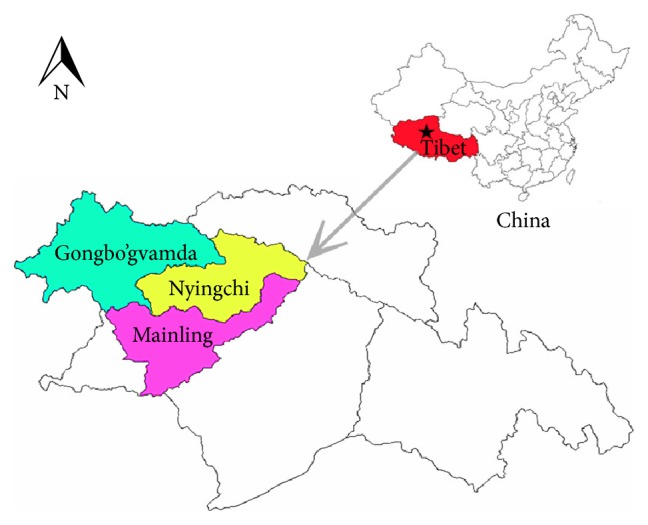
The map of geographical distribution of* Cysticercus tenuicollis* serological investigation in Tibet.

This study was conducted in three counties (Nyingchi, Mainling, and Gongbo'gvamda) of Tibet, China, and samples were collected from pigs at different slaughterhouses in these three counties that have an average elevation of 3100 meters with the largest continuous high elevation ecosystem (Figure 1).

### 2.2. Samples Collection and Processing

**Figure 2:**
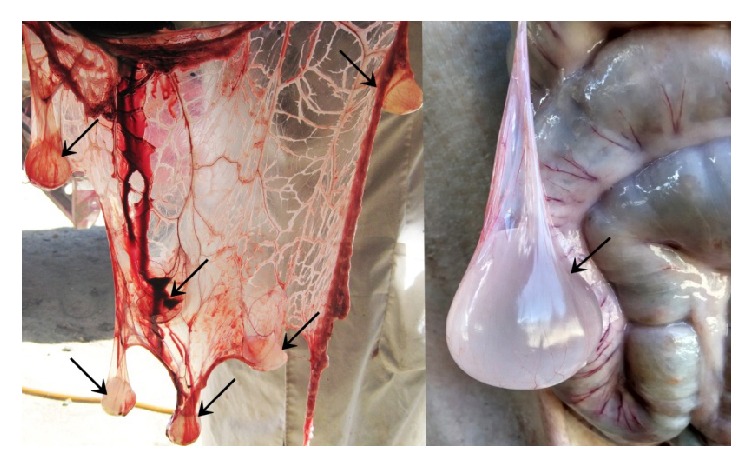
*Cysticercus tenuicollis* infection in clinical autopsy in Tibetan pigs, China.

In present study, a total of 112 samples were collected from Tibetan pigs (independent of age, sex, and breed by using simple random technique for sample collection) at different slaughterhouses of Nyingchi, Mainling, and Gongbo'gvamda of Tibet, in 2014. The information regarding each sample was captured on a prescribed performa. Similarly, a total of 86 samples were also collected from these counties in 2015. After autopsy, each positive sample was recorded and collected (Figure 2). The larval stages were washed extensively in 0.9% sodium chloride solution and identified through morphological examinations. Subsequently, they were fixed in 75% alcohol (V/V) and stored at −20°C, until subsequent use and further analysis. Total DNA of the larvae of* Cysticercus tenuicollis* was extracted using the commercial kit (TIANamp Genomic DNA Kit, TianGen, China), according to manufacturer's instructions. The eluted DNA was stored at −20°C until further use.

A PCR amplification approach was used to amplify a fragment (~560 bp) of the COX2 gene. For this purpose polymerase chain reaction primer forward GAAGTTGGTTACTGAAAAG and reverse ATTCCATGATTAACACCAC were used with the following cycling parameters: 35 cycles at 95°C for 30 seconds and 72°C for 1 minutes, annealing at 55°C for 30 seconds, and final extension at 72°C for 5 minutes. ddH_2_O was used as blank control group. PCR products were separated on agarose gel (1.0%) along with ethidium bromide (at the rate of 0.5 *μ*g/ml) and electrophoresis was performed on 0.5x TBE buffer at 5 V/cm for 60 minutes. The products were purified using a TaKaRaMiniBEST Agarose Gel DNA Extraction Kit Ver.4.0 (Takara Biotechnology Co., Ltd., Dalian, China), according to manufacturer's instructions. The obtained positive products were sequenced by a commercial company (Quintara Biosciences, Wuhan, China).

### 2.3. Phylogenetic and Sequence Analysis

The nucleotide sequences of the COX2 gene of* Cysticercus tenuicollis* were compared with previously reported* Cysticercus tenuicollis* sequences available at NCBI database (appears in Table 2). Multiple alignments and phylogenetic analysis were conducted using Molecular Evolutionary Genetic Analysis (MEGA 6.0) software. Phylogenetic tree was constructed using the neighbor-joining method. The evolutionary distances were estimated using the Kimura two-parameter method. A bootstrapping test was performed with 1000 duplicates and the transversion/transition rate was set at 2.0.

### 2.4. Statistical Analysis

The prevalence of* Cysticercus tenuicollis* in Tibetan pigs relating to different genders and growing stages was analyzed statistically by using Chi-square test, while 95% CI and odd ratio were also determined. The value of *P* < 0.05 was considered as statistically significant.

## 3. Results

### 3.1. Prevalence of* Cysticercus tenuicollis*

The results showed the highest infection rate of* Cysticercus tenuicollis *in Tibetan pigs was 43.93% in all three counties. The year wise prevalence of* Cysticercus tenuicollis* was 42.86% and 45.35% in 2014 and 2015, respectively. The sex wise prevalence of* Cysticercus tenuicollis* was 42.37% and 44.68% in male and 46.15% and 43.40% in female pigs in 2014 and 2015, respectively, while during different growing stages of Tibetan pigs, the prevalence was 29.51%, 60.00%, and 56.25% in 2014 and 31.43%, 69.57%, and 42.86% in 2015 in juveniles, subadults, and adults, respectively. The rate of infection among males and females was not found statistically significantly (*P* > 0.05); however, a significant difference (*P* < 0.05) was observed among different growing stages of Tibetan pigs as shown in Table 1.

### 3.2. Phylogenetic Analysis

**Figure 3:**
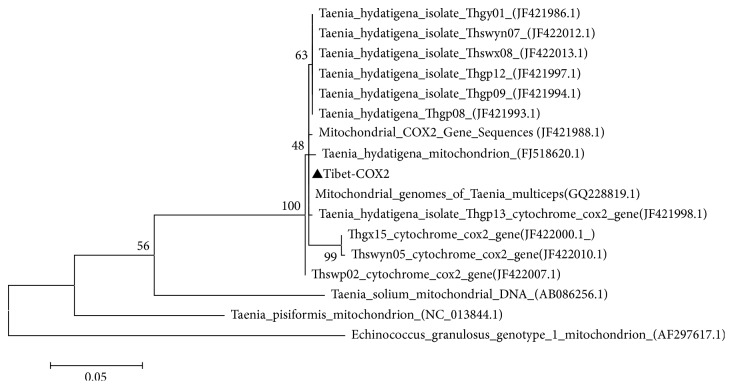
Phylogenetic tree constructed by the neighbor-joining method in MEGA 6.0, using nucleotide sequences of the COX2 gene (Outgroup AF297617.1).

**Figure 4:**
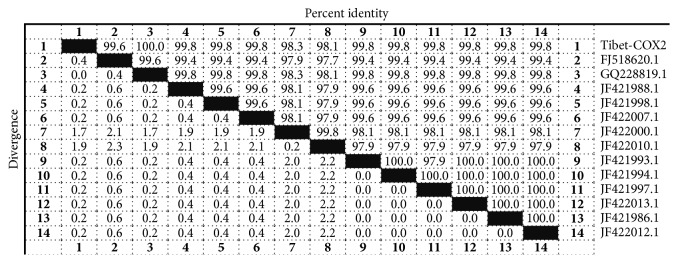
Homology comparison of nucleotide sequences of the isolates strains with previously reported strains (100%).

To determine the evolutionary relationships between the larvae stage of* Cysticercus tenuicollis* (named: Tibet-COX2) and previously reported 16 reference strains, the result revealed that the nucleotide sequence of the Tibet-COX2 strain was 97.7%–100% identical to that of the previously reported strains (accession numbers GQ228819.1, JF421988.1, JF421998.1, JF422007.1, FJ518620.1, JF422000.1, JF421986.1, JF422012.1, JF421997, JF422013.1, JF421994.1, JF421993.1, JF422010.1, and AB086256.1, NC-013844.1, and AF297617.1) (Table 2). The phylogenetic analysis was performed based on the COX2 gene (Figure 3). As determined by the phylogenetic tree, the homology of the nucleotide sequence between the Tibet-COX2 strain and some reference strains (GQ228819.1, JF421988.1, JF421998.1, JF422007.1, FJ518620.1, JF422000.1, JF421986.1, JF422012.1, JF421997, JF422013.1, JF421994.1, JF421993.1, and JF422010.1) illustrated a close homology. The result showed that the Tibet-COX2 strain was very closely related to reference strains isolated from Gansu, Hunan, and Sichuan provinces, which are very close regions to Tibet (Figure 3). In addition, the nucleotide sequences of isolated strains shared 97.7%–100% sequence identify with the reference strains (Figure 4).

## 4. Discussion

*Cysticercus tenuicollis* is the larvae stage of* Taenia hydatigena,* which is a common parasite of pigs, cattle, buffalo, yak, sheep, goat, camel, horse, and human [6]. Many countries in the world have reported* Cysticercus tenuicollis* since its first report [4, 6, 7]. This disease remains a severe problem in pigs especially under the age of one year, where there are no effective measures that result in very high mortality. China is the largest pigs producing country, while parasitosis has become a major threat to pigs industry in the country, which causes serious illness [13]. As the only plateau and alpine pasture of pig breeds in China,* Cysticercus tenuicollis* has not yet been reported in Tibetan pigs. The recent research showed that a high infection rate of* Cysticercus tenuicollis* was found in Tibetan pigs, which is higher than that reported in pigs from Dali (6.06%) and Pingle (1.11%) [13, 14]. The prevalence difference of* Cysticercus tenuicollis* in our study and the previous reports may be due to the differences in free-range feeding pattern, ecological environment, and existence of the wild dogs, as well as the knowledge level of the breeder [15]. During the survey, we found that the local people were not aware of using antiparasitic drugs, secondly improperly or frequently using the antimicrobial agents. So, it may be the possible reason for the high prevalence rate of* Cysticercus tenuicollis.* Tibetan pigs are mainly fed by the extensive breeding environment combined with the dry lot husbandry [16]. Under the special breeding system, this animal get more chance to expose to the external environment; among them, subadults and adults have the longest time of outdoor activities which increases a higher infection risk of infecting the* Cysticercus tenuicollis*, compared with the juveniles Tibetan pigs [6].

Currently studies have showed that the mitochondrial genome structure of Tapeworms is similar to other mitochondrial genomes of eukaryotes that include 12 genes encoding proteins [17]. Among them, the mitochondrial gene cytochrome oxidase subunit 2 was the ideal genetic markers, which was used to make phylogenetic analysis [18]. In our study, the isolated parasite was phylogenetically compared to reference parasite, based on the COX2 gene coding sequence (CDS). The phylogenetic analysis demonstrated that the isolates share a close homology with some reference strains isolated in Gansu, Hunan, and Sichuan. In addition, the nucleotide sequences of the isolated strains shared 97.7%–100% sequence identify with the reference strains.

In conclusion, the present survey revealed the high prevalence of* Cysticercus tenuicollis* infection in Tibetan pigs; herein, we suggest some measures to prevent and control this infection. At first, anthelmintics must be an important practice to prevent the infection and minimize the serious economic losses. Secondly, it is important to improve the management of swine breeding to reduce the spread of disease through direct contact between dogs and Tibetan pigs. Additionally, practice of undercooked animal innards to dogs in study area should be avoided. Finally, it is necessary to change the husbandry of free-range into captivity system in these rural areas.

## Figures and Tables

**Table 1:** Seroprevalence of *Cysticercus tenuicollis* in Tibetan pigs in different gender and growing stage in Tibet in 2014 and 2015.

Variable	2014		2015		Total		95% CI	Odd ratio/*P* value
	Number of pigs	Number of positive samples (%)	Number of pigs	Number of positive samples (%)	Number of pigs	Number of positive samples (%)		
Gender								
Male	59	25 (42.37)	47	21 (44.68)	106	46 (43.39)	34.20–52.95	OR = 0.95 [reciprocal = 1.05]
Female	53	23 (43.40)	39	18 (46.15)	92	41 (44.56)	34.65–54.81	
Total	112	48 (42.86)	86	39 (45.35)	198	87 (43.93)	37.14–50.92	—
Growing stage								
Juveniles	61	18 (29.51)	35	11 (31.43)	96	29 (30.20)	21.66–39.93	Mantel-Haenszel
Subadults	35	21 (60.00)	23	16 (69.57)	58	37 (63.79)	50.88–75.36	Chi-sq *P* < 0.008
Adults	16	9 (56.25)	28	12 (42.86)	44	21 (47.27)	33.35–62.39	
Total	112	48 (42.86)	86	39 (45.35)	198	87 (43.93)	37.14–50.92	—

**Table 2:** The data obtained from NCBI for analysis in this study.

Accession	Regions	Year	Species	Strain
GQ228819.1	Gansu	2010	Sheep	IU
JF421988.1	Sichuan	2012	Goat	Thgj03
JF421998.1	Sichuan	2012	Goat	Thgp13
JF422007.1	Sichuan	2012	Pig	Thswp02
FJ518620.1	Hunan	2011	Human	IU
JF422000.1	Sichuan	2012	Goat	Thgx15
JF422010.1	Sichuan	2012	Pig	Thswyn05
JF421993.1	Sichuan	2012	Goat	Thgp08
JF421994.1	Sichuan	2012	Goat	Thgp09
JF421997.1	Sichuan	2012	Goat	Thgp12
JF422013.1	Sichuan	2012	Pig	Thswx08
JF421986.1	Sichuan	2012	Goat	Thgy01
JF422012.1	Sichuan	2012	Pig	Thswyn07
